# Quasi-Simultaneous Detection of Ammonia and Nitrous Oxide by Photoacoustic Phase-Resolved Method: A Proof-of-Concept

**DOI:** 10.1021/acs.analchem.5c04976

**Published:** 2025-12-10

**Authors:** Daniel da Silva Santos, Leonardo Mota, Guilherme Rodrigues Lima, Eduardo César da Matta, Thiago Lemos Alvarenga, Marc-Simon Bahr, Israel Andrade Esquef, Marcus Wolff, Marcelo Gomes da Silva

**Affiliations:** † 28109 Universidade Estadual do Norte Fluminense Darcy Ribeiro, Av. Alberto Lamego, 2000, Campos dos Goytacazes 28013-602, Rio de Janeiro, Brazil; ‡ 28126 Universidade Federal do Espírito Santo, Alto Universitário, s/n, Alegre 29500-000, Espírito Santo, Brazil; § Heinrich Blasius Institute of Physical Technologies, Hamburg University of Applied Sciences, Hamburg 20999, Germany

## Abstract

This work presents a method for the quasi-simultaneous detection of trace gas molecules using photoacoustic spectroscopy with phase-resolved method. By utilizing two lasers to excite ammonia (NH_3_) and nitrous oxide (N_2_O) in a mixture and employing phase-sensitive detection, the individual contributions of these gases were separated. The separation was achieved by adjusting the phase modulation of the N_2_O exciting laser until a 90-degree phase difference was established between its signal and that of NH_3_. The results demonstrate the absence of spectral overlap between the two compounds, enabling independent and quasi-simultaneous detection. Calibration curves highlighted the behavior and cross-interference between NH_3_ and N_2_O. For NH_3_, linear profiles with regression coefficients *R* = 0.998 and *R* = 0.999 were observed, when N_2_ or N_2_ + N_2_O were used as diluent gases, respectively. The corresponding sensitivities were (3.0 ± 0.1) μV·ppmv^–1^and (3.10 ± 0.08) μV·ppmv^–1^. The lower detection limits (LDLs) were 51 ppbv and 67 ppbv, respectively. For N_2_O, calibration data were similarly robust with LDLs of 262 ppbv and 255 ppbv when diluted with N_2_ and N_2_ + NH_3_, respectively. Minimal cross-interference effects were found, confirming the selectivity and reliability of this method for quantifying NH_3_ and N_2_O in gas mixtures. The technique offers advantages over other methods and is applicable to studying emissions related to agriculture with nitrogen fertilizers.

## Graphical abstract

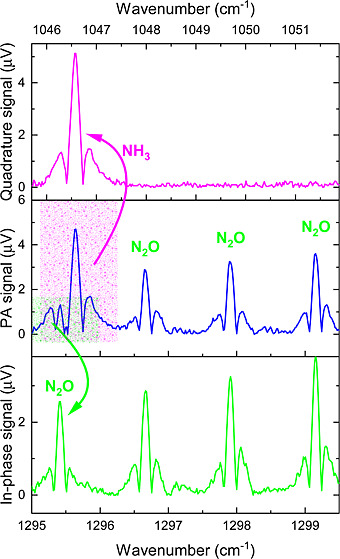

## Introduction

Spectroscopic techniques have been successfully employed for gas detection in the near- and mid-infrared regions paired with solid-state radiation sources such as quantum cascade lasers (QCLs),^1^ interband cascade lasers (ICLs),^2^ laser-diode-pumped crystals,^3^ and optical parametric oscillator.^4^ Among these techniques, photoacoustic spectroscopy (PAS) is particularly notable for trace-level gas sensing because of its high sensitivity, selectivity, and real-time measurement capability. Progress includes the development of new devices equipped with sensitive microphones,^5^ cantilever-enhanced,^6^ or quartz-enhanced PAS (QEPAS).^7–10^ Besides, the fabrication of cost-effective PA cells achieved through 3D printing technologies has appeared.^11^

Simultaneous detection of multiple gases is of paramount importance in environmental monitoring, industrial safety, and medical diagnostics, where rapid and precise multicomponent analysis enables real-time decision-making and improves process efficiency. For instance, QCL-based dual-spectroscopy systems have enabled simultaneous monitoring of CO, N_2_O, and H_2_O with high spectral resolution and rapid acquisition rates.^12^ Self-calibrated 2*f*/1*f* wavelength modulation spectroscopy techniques achieve inherent calibration and stability, minimizing errors associated with laser intensity fluctuations.^13^ Furthermore, quartz crystal tuning fork-enhanced laser spectroscopy has shown noteworthy performance for simultaneous detection of three species (e.g., H_2_O, CO_2_, and CH_4_) with ppmv sensitivity and compact configuration.^14^

Recent advancements in PAS research have focused on the “simultaneous detection” of multiple molecules, with frequency division multiplexing (FDM) and time division multiplexing (TDM) emerging as the most widely used methods. Nevertheless, while TDM requires modulation at different frequencies in a temporal sequence or alternation between radiation sources, FDM signals may experience greater attenuation, leading to uneven sensitivity, which could be problematic.^15–21^ Yet, there is a lack of literature that addresses the temporal resolution of “simultaneous” measurements in a single resonator.^22,23^ This highlights the complexity of utilizing multiple laser sources operating at the same resonance frequency within a straightforward experimental setup.

A related technique, phase-resolved method (PRM) relies on the assumption that distinct absorbing centers are present, which inherently entails a phase difference between them due to different nonradiative and thermal relaxation times (τ and τ_β_). This approach has been shown to work effectively for solid, liquid and biological samples.^24–28^ However, a central challenge in PA-based simultaneous gas detection still remains: extracting overlapping signals when molecular relaxation times are nearly identical.

The very first use of phase-sensitive detection to distinguish gas molecules in multicomponent mixtures was reported by Kosterev et al., in 2004.^29^ They employed a QEPAS-based system operating at about *f* = 32.7 kHz, a reduced pressure (50 Torr) and a second-harmonic wavelength-modulated (WM-2*f*) QCL as a radiation source – such conditions ensure high sensitivity for QEPAS. Moreover, since τ is longer at lower pressures, the authors stated that an observable phase is detected when the condition 2π*f* ≥ τ^–1^ is fulfilled. Hence, they applied PRM to detect carbon monoxide merged in propene. To the best of our knowledge, though under very specific circumstances, this study remains the sole instance to date that addresses the application of PRM in gaseous molecules. The breakthrough of our work consists of electronically phase-shifting the QCLs’ wave modulation to introduce a controlled (measurable) phase lag between the generated PA signals. This framework allowed us to completely separate the absorption spectra of NH_3_ and N_2_O from a single quasi-simultaneous measurement of a gas mixture.

### Theoretical Aspects of Phase-Resolved Method

For a simplified approach, when heat diffusion and dynamic viscosity are neglected, Morse and Ingard derived the inhomogeneous wave equation that relates the acoustic pressure *p* and the heat source *H* ^30^

1 $$\nabla^{2}p-\frac{1}{c^{2}}\frac{\partial^{2}p}{\partial t^{2}}=-\frac{\gamma -1}{c^{2}}\frac{\partial H}{\partial t}$$

where *c* is the sound speed and γ is the ratio of specific heats of the gas for constant pressure and volume. Equation 1 is solved by considering the periodic heating (ω) induced by photon absorption in the gas species, and can be expressed as a superposition of normal acoustic modes *p*_*k*_(r,ω). The solution to eq 1 for the case of cylindrical geometry is provided elsewhere.^31,32^

All measurements reported in this work were conducted using the first longitudinal resonance mode of the photoacoustic cell (*k* = 1). Under this condition, the photoacoustic signal can be expressed by eq 2

2 $$S\left(ṽ\right)=p_{1}\left(r_{mic}{\mathrm{,\omega}}_{1}\right)R_{mic}=C_{cell}\cdot P\left(ṽ\right)\cdot N\cdot \sigma \left(ṽ\right)$$

where *R*_mic_ is the microphone sensitivity given in millivolts per pascal; P(ṽ) is the laser power at ṽ ; *N* is the density of the absorbing molecules; σ(ṽ) is their absorption cross-section at ṽ ; and *C*_cell_ denotes the cell constant, which depends on both the microphone sensitivity and the geometrical configuration of the cell.^31^

When the laser radiation at wavenumber ${ṽ}_{0}$ passes through the absorbing medium, a fraction of the optical power is converted into heat through nonradiative relaxation processes. The absorbed power per unit volume, *H*, is therefore proportional to the number density of absorbing molecules *N*, the absorption cross section $\sigma \left({ṽ}_{0}\right)$ , and the laser power $P\left({ṽ}_{0}\right)$ at the corresponding wavenumber. By varying the laser temperature to tune the central wavenumber ${ṽ}_{0}$ , the molecular absorption spectrum is obtained.

In wavelength modulation spectroscopy, the oscillation of the emission wavenumber around its central value ${ṽ}_{0}$ is responsible for generating the photoacoustic signal. Then, for small amplitude oscillations of ${ṽ}_{0},\delta ṽ={\frac{\partial ṽ}{\partial i}|}_{{ṽ}_{0}}\delta i={\frac{\partial ṽ}{\partial i}|}_{{ṽ}_{0}}\delta i_{0}\exp\left[j\left(\omega t+\phi \right)\right]$ where δ*i*_0_ is the amplitude of the laser current modulation, and ω denotes the angular modulation frequency (2π*f*). Under this assumption, the absorption cross section can be expanded in a Taylor series (eq 3)­

3 $$\sigma \left(ṽ\right)=\sigma \left({ṽ}_{0}\right)+{\frac{\partial \sigma }{\partial ṽ}|}_{{ṽ}_{0}}\delta_{{ṽ}_{0}}\left(i\right)e^{j\left(\omega t+\phi \right)}+\frac{1}{2}{\frac{\partial^{2}\sigma }{\partial {ṽ}^{2}}|}_{{ṽ}_{0}}\delta_{{ṽ}_{0}}^{2}\left(i\right)e^{2j\left(\omega t+\phi \right)}+\cdot \cdot \cdot$$

where ${\frac{\partial ṽ}{\partial i}|}_{{ṽ}_{0}}{\delta i}_{0}=\delta_{{ṽ}_{0}}$ .

Since the second harmonic of the wavelength modulation was utilized (third term of eq 3), the laser was modulated at ω_1_/2, and the lock-in detection was performed at 2ω_1_, thereby enabling the measurement of a derivative photoacoustic signal at the resonance frequency of the photoacoustic cell.

For a sample containing two types of target molecules excited with their respective appropriate radiation sources, the total photoacoustic signal can be expressed by eq 4 below, derived from eqs 2 and 3:

$$S_{{ṽ}_{0}}\left(t\right)=S_{{ṽ}_{0},N_{2}O}\left(t\right)+S_{{ṽ}_{0},{NH}_{3}}\left(t\right),S_{{ṽ}_{0}}(t)=C_{cell}[N_{N_{2}O}P_{N_{2}O}\left({ṽ}_{0,N_{2}O}\right)\sigma_{N_{2}O}\left({ṽ}_{0,N_{2}O}\right)+N_{{NH}_{3}}P_{{NH}_{3}}\left({ṽ}_{0,{NH}_{3}}\right)\sigma_{{NH}_{3}}\left({ṽ}_{0,{NH}_{3}}\right)]=\frac{C_{cell}}{2}\left(N_{N_{2}O}P_{N_{2}O}\left({ṽ}_{0,N_{2}O}\right){\frac{\partial^{2}\sigma }{\partial {ṽ}^{2}}|}_{{ṽ}_{0,N_{2}O}}\delta_{{ṽ}_{0,N_{2}O}}^{2}e^{2j\left(\omega_{1}t+\phi_{N_{2}O}\right)}+N_{{NH}_{3}}P_{{NH}_{3}}\left({ṽ}_{0,{NH}_{3}}\right){\frac{\partial^{2}\sigma }{\partial {ṽ}^{2}}|}_{{ṽ}_{0{NH}_{3}}}\delta_{{ṽ}_{0{NH}_{3}}}^{2}e^{2j\left(\omega_{1}t+\phi_{{NH}_{3}}\right)}\right)$$ 4

where $\phi_{N_{2}O}$ and ϕ_NH_3__ are the phases of the photoacoustic signals of these molecules relative to the excitation source.

When two absorbing centers exhibit similar phases, the direct application of PRM becomes challenging. However, an artificial phase difference (Δϕ = θ_FG_ ≠ 0) can be introduced by adding a phase shift to the modulation of one of the radiation sources (eq 5):

5 $$S_{{ṽ}_{0}}\left(t\right)=S_{{ṽ}_{0},N_{2}O}e^{2j\left(\omega_{1}t+\phi_{N_{2}O}+\theta_{FG}\right)}+S_{{ṽ}_{0},{NH}_{3}}e^{2j\left(\omega_{1}t+\phi_{{NH}_{3}}\right)}$$

here, $S_{{ṽ}_{0,m}}=\frac{C_{cell}}{2}N_{m}P_{m}\left({ṽ}_{0,m}\right){\frac{\partial^{2}\sigma }{\partial {ṽ}^{2}}|}_{{ṽ}_{0,m}}\delta_{{ṽ}_{0,m}}^{2}$ , where the subscript *m* refers to the target gas, which can be either N_2_O or NH_3_, represents the amplitudes of the N_2_O and NH_3_ signals; and θ_FG_ is the phase increment applied to the N_2_O excitation, set using a function generator.

### Experimental Details

The PA experimental arrangement is depicted in Figure 1. This setup includes two radiation sources: one for detecting NH_3_ within the spectral range from 1045.70 to 1051.88 cm^–1^ (Thorlabs, ICL-QD9556HHLH-A) and another for detecting N_2_O in the spectral range from 1295.00 to 1299.50 cm^–1^ (Thorlabs, QCL-QD7716HH). The wavenumber scan was obtained by varying the temperature of N_2_O laser holder between 15 °C (53 mW) and 45 °C (34.4 mW) and that of NH_3_ laser holder between 20 °C (88.0 mW) and 50 °C (67.0 mW), at a temperature rate of 0.05 °Cs^–1^. The wavenumber-to-temperature tuning rates were 0.150 cm^–1^/°C and 0.206 cm^–1^/°C for N_2_O and NH_3_, respectively. To prevent laser damage due to cross-interference, each beam was slightly tilted and blocked to avoid entering the opposite laser. Two power supplies (Thorlabs, ITC4002QCL) individually powered the lasers.

**Figure 1:**
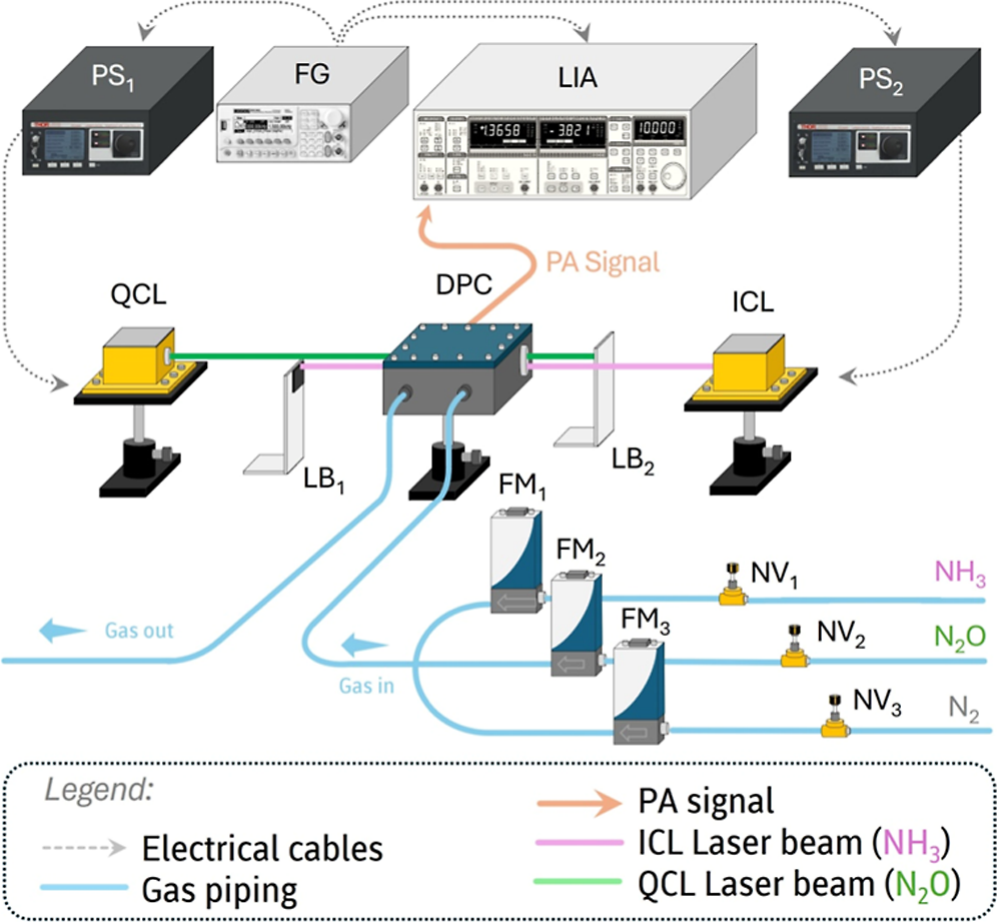
Experimental apparatus dedicated to quasi-simultaneous measurements of NH_3_ and N_2_O. The acronyms are defined as PS (Power Supply), FG (Function Generator), LIA (Lock-In Amplifier), DPC (Differential Photoacoustic Cell), LB (Laser Block), FM (Flow Meter) and NV (Needle Valve).

The gas sensor consists of a differential photoacoustic cell (DPC) with the first longitudinal (out-of-phase ring resonance^11^) mode at 3.9 kHz and a quality factor of about 15. It is equipped with two microphones to detect the photoacoustic signal.^11^ The microphone signals are amplified using a digital lock-in amplifier (Stanford Research SR830), set to a time constant of 0.3 s and employing a second-order low-pass filter (12 dB roll-off). A sampling rate of 0.5 Hz was established in the Python-written data acquisition program.

Second-harmonic wavelength modulation (WM-2f) was achieved by superimposing a sinusoidal AC current with a peak-to-peak amplitude of 8 mA on a DC bias current of 295 mA for N_2_O and on a DC bias current of 520 mA for NH_3_. A function generator with two synchronized output channels (RIGOL DG 1022) was employed.

The AC frequency was adjusted to be half of the DPC’s resonance frequency (*f*/2), and the lock-in amplifier was configured to measure the second harmonic (*f*).^33^ The output channel of the function generator used for the modulation of N_2_O allows phase increments relative to that of the other channel, enabling the temporal separation of the photoacoustic signals from both molecules on a measurable scale. The photoacoustic signal *S* as a function of time is given by eq 5.

Starting from certified standard samples of 5.0 ppmv of N_2_O in nitrogen and 10 ppmv of NH_3_ in nitrogen, both supplied by White Martins Ltd., different diluted concentrations of the two species were prepared by mixing them with each other or with pure nitrogen (99.9%). The dilution ratios were manually adjusted using mechanical valves and monitored with mass flow meters (Alicat Scientific, M-500SCCM-D/5M). For all mixtures, the total gas flow rate was maintained at 300 sccm.

## Results and Discussion

The WM-2f PA spectra of a gas mixture containing 4.67 ppmv NH_3_ (140 sccm) and 2.67 ppmv N_2_O (160 sccm) are shown in Figure 2. The central panel presents the original PA spectrum obtained when both gas species were excited simultaneously. In the first half of the spectrum, an overlap between the absorption lines of N_2_O and NH_3_ occurs. To resolve these features, the PRM technique was applied. Prior to this, since the laser drivers were modulated by a separate output of a dual-channel function generator, a relative time shift between the N_2_O and NH_3_ laser current modulations was established by setting the phase difference between the channels to θ_FG_ = 15°, 24.3°, and 45°. In this configuration, a phase shift θ_FG_ corresponds to a time delay of θ_FG_/360° × (2/3.9 kHz), where 3.9 kHz represents the resonance frequency of the photoacoustic cell. In other words, a phase shift of θ_FG_ = 15° corresponds to 15°/360° × (2/3.9 kHz) = 21.4 μs; θ_FG_ = 24.3° corresponds to 34.6 μs; θ_FG_ = 45° corresponds to 64.1 μs.

**Figure 2:**
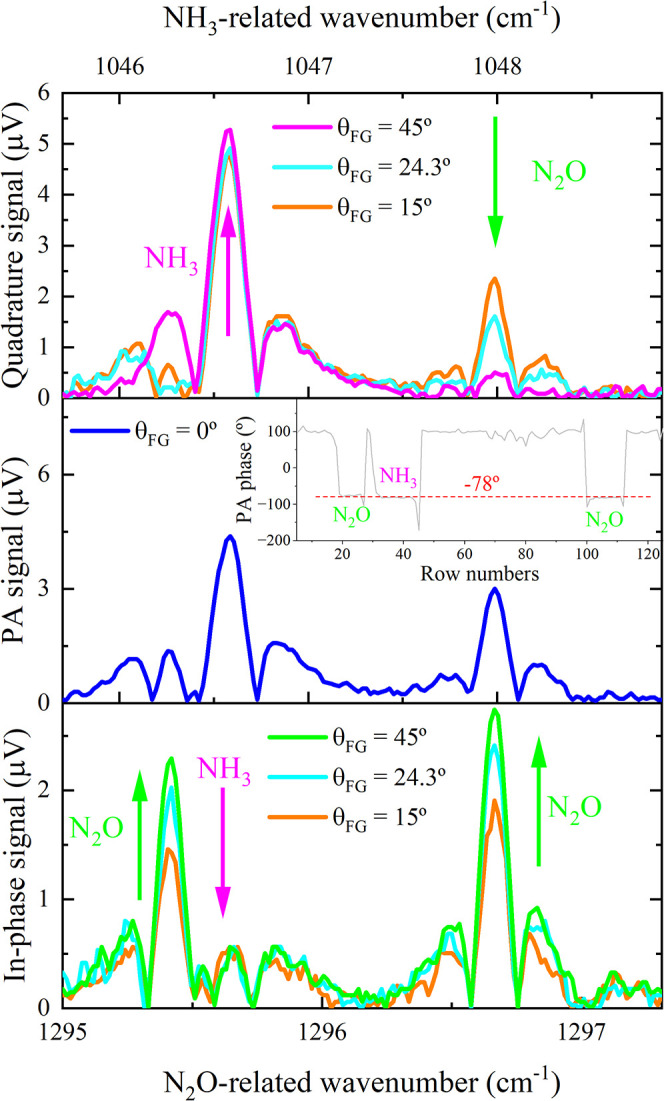
WM-2f PA spectra as a function of wavenumber. Central panel is the measured spectrum (θ_FG_ = 0°) of a gas mixture consisting of 4.67 ppmv NH_3_ and 2.67 ppmv N_2_O. The upper and bottom panels refer, respectively, to quadrature and in-phase spectra of the same gas mixture for different θ_FG_ values. The inset figure shows that ${2\phi }_{N_{2}O}$ and 2ϕ_NH_3__ (in eq 5) are nearly identical, both are approximately −78°.

The upper and lower panels depict the quadrature and in-phase PA signals as functions of θ_FG_, indicating that an increase in θ_FG_ results in an improved spectral resolution. For θ_FG_ = 45°, the projection of the NH_3_ signal is predominantly aligned with the lock-in *Y*-axis (top panel, magenta line). In contrast, the signal derived from N_2_O remained nearly constant along the lock-in *X*-axis (bottom panel, green line). Nonetheless, cross-talk between N_2_O and NH_3_ persists, as indicated by a green arrow in the top graph and a magenta arrow in the bottom graph.

Starting from a common natural photoacoustic phase shift of (−78 ± 6)°, this effect is illustrated in Figure 3a–d, wherein for each adjusted value of θ_FG_ on the function generator, the induced phase lag between the PA phasors of NH_3_ (solid magenta arrow) and N_2_O (dashed green arrow) was twice as large (eq 5). Thus, PRM was applied. To do so, the starting point was that when θ_FG_ = 45°, which implied an effective separation equal to 90°.

**Figure 3:**
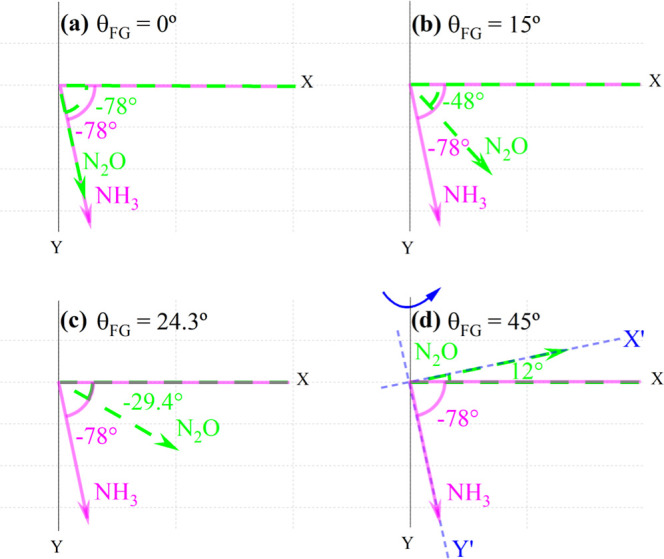
Phasor diagram of the PA signal for NH_3_ and N_2_O (dashed green line) as a function of θ_FG_. From (a to d), θ_FG_ were set to be 0°, 15°, 24.3° and 45°, respectively. In (d), the short-dashed blue line illustrates the rotation of the reference frame, enabling full isolation of the PA spectra of NH_3_ along the *Y*′-axis and N_2_O along the *X*′-axis.

Figure 4 portrays the resulting quasi-simultaneous detection of 4.67 ppmv NH_3_ and 2.67 ppmv N_2_O. The shaded areas in the middle panel highlight the region where a molecular superposition is clearly observed. From a phasor perspective, it corresponds to the scenario presented in Figure 3a. To achieve complete spectral resolution between NH_3_ and N_2_O, the initial configuration was as outlined in Figure 3d, i.e., θ_FG_ = 45°. From this, progressive rotations of the lock-in reference frame (denoted by the rounded blue arrow) were performed to ascertain a novel arrangement that would allow complete separation of the PA spectra of NH_3_ and N_2_O. This condition was successfully realized when the reference frame was rotated by 12° (short-dashed blue line). As a result, the WM-2f PA spectrum of NH_3_ was entirely phase-resolved in the quadrature component (upper panel of Figure 4), while the in-phase component unequivocally exhibited the characteristic WM-2f PA spectrum of N_2_O (bottom panel of Figure 4).

**Figure 4:**
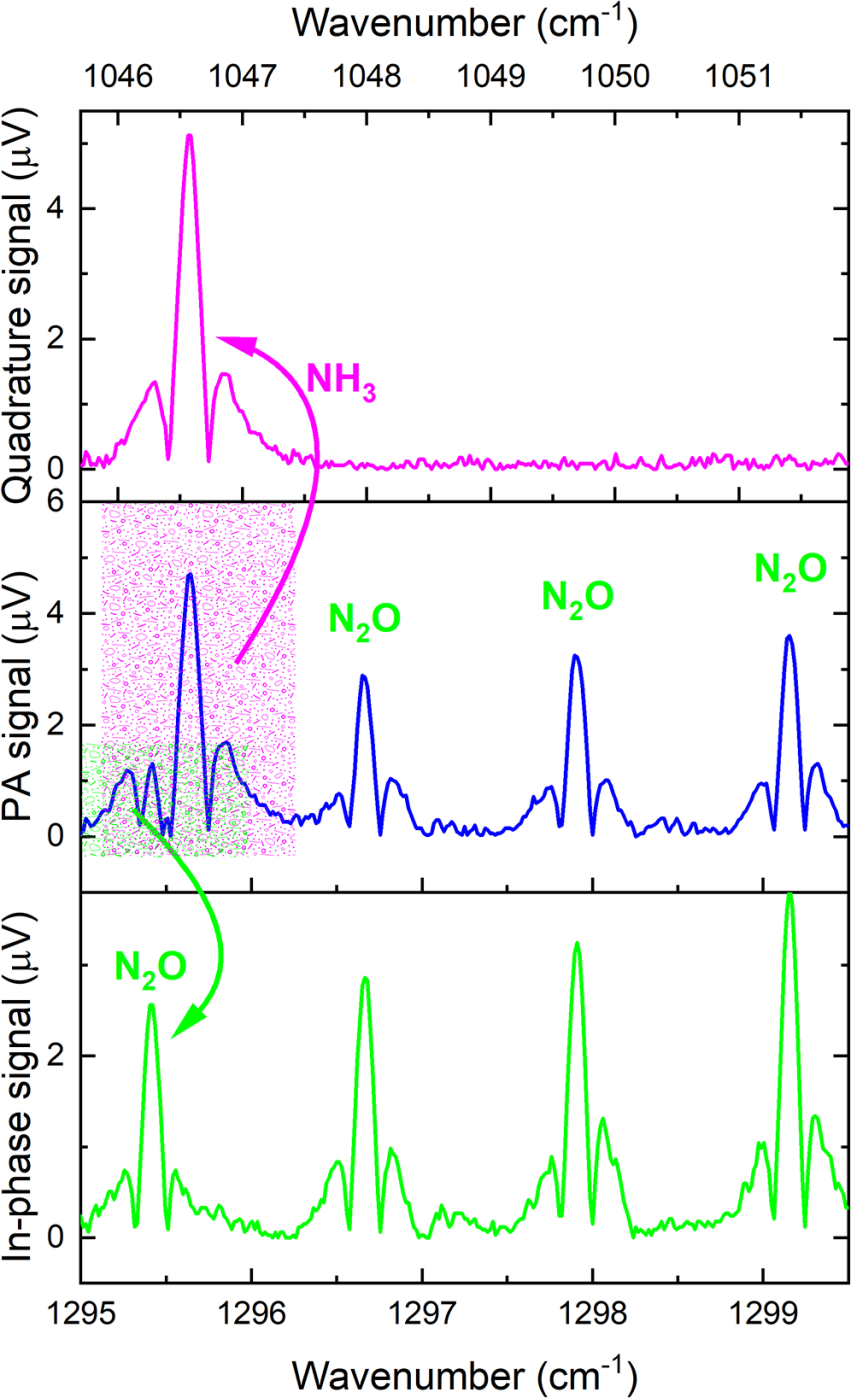
Resulting PA–PRM for NH_3_ and N_2_O. The center panel shows the as measured PA spectrum of a mixture containing 4.67 ppmv NH_3_ plus 2.67 ppmv N_2_O. The highlighted areas in magenta and green display overlapped lines of NH_3_ and N_2_O. The upper and bottom panels correspond, respectively, to the quasi-simultaneously measured quadrature and in-phase spectra taking into account a θ_FG_ = 45° combined with a 12° lock-in phase increment (rotation of the reference frame).

### Calibration Curves: Phase Behavior, Sensitivity, Detection Limit and Cross-Talk between NH_3_ and N_2_O

Figure 5 displays the PA phases as functions of N_2_O and NH_3_ concentrations. Each data point represents the average of 300 measurements (inset plot in Figure 5), and the error bar corresponds to their standard deviation. The PRM was applied by initially setting the signal phase to −90° when only 2.0 ppmv of NH_3_, diluted in nitrogen and free from N_2_O, was flowing through the PA cell. The same approach was applied for 4.0 ppmv N_2_O in nitrogen, free from NH_3_, with its phase set to 0°. Subsequently, in separate experiments, further dilutions of both gases in nitrogen were prepared, and the signal phases for each molecule at four new concentrations were measured (open blue circles for N_2_O and red triangles for NH_3_, Figure 5). In a second experiment, involving mixtures of NH_3_, N_2_O, and N_2_, the gas components were combined in flow to reproduce the same concentrations of N_2_O and NH_3_ as in the previous experiment. The phase values of the signal for each gas mixture are plotted as black filled circles in Figure 5. Consequently, the PA signal phase varies according to the relative contributions of both molecular species. This trend is illustrated by the black points in Figure 5, where the PA phase approaches 0° as the N_2_O concentration increases to 4 ppmv in the absence of NH_3_, and approaches −90° when the NH_3_ concentration reaches 2 ppmv in the absence of N_2_O. These results strongly support the effectiveness of phase separation and gaseous detection across different concentrations using the proposed approach.

**Figure 5:**
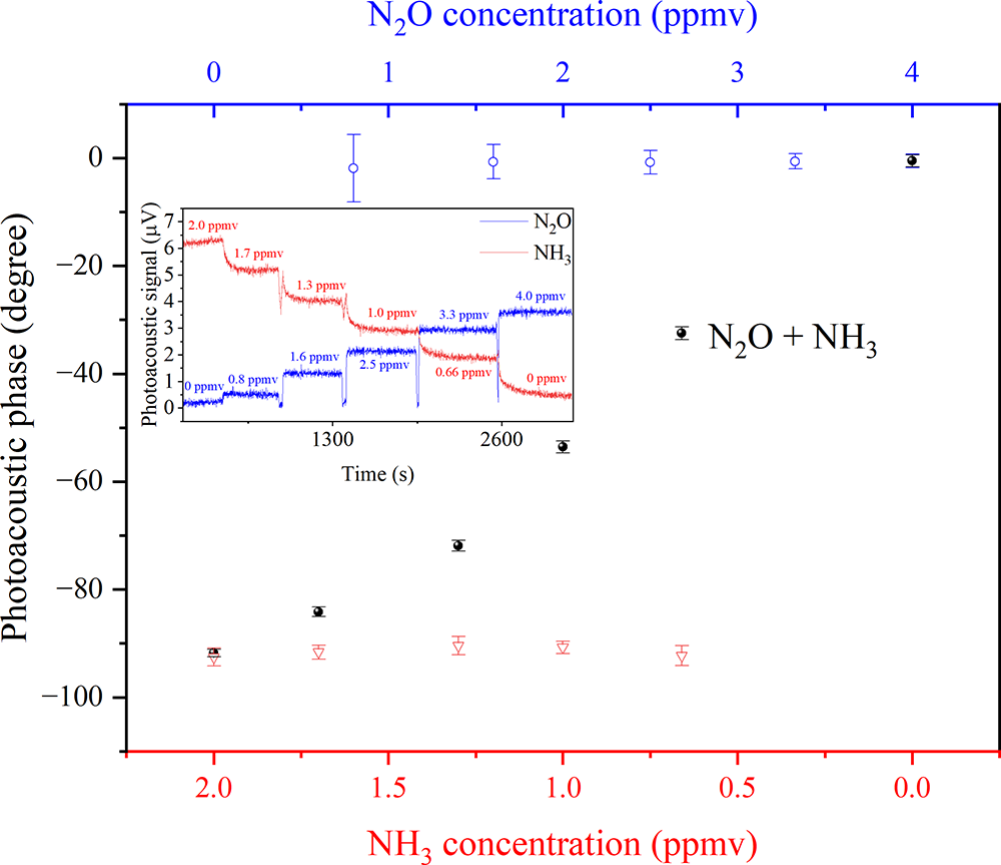
Photoacoustic phase response as a function of gas concentration measured by the PRM. The top horizontal axis shows the concentration of N_2_O in N_2_, while the bottom horizontal axis shows the concentration of NH_3_ in N_2_. The vertical axis represents the photoacoustic phase. Blue symbols correspond to the phase of N_2_O measured separately at different concentrations in N_2_. Red symbols represent the phase of NH_3_ measured separately at different concentrations in N_2_. Black symbols indicate the phase of the gas mixture during simultaneous dilution of NH_3_ and N_2_O in N_2_. The inset displays the quadrature signal of NH_3_ (red) and the in-phase signal of N_2_O (blue) in the PRM measurement of the mixture, illustrating the phase differentiation between the two gases.

With the aim of investigating both the PRM performance and the occurrence of cross-talk between N_2_O and NH_3_, Figure 6 depicts the calibration curves (open symbols), while the solid lines represent the best linear fits to the experimental data. In Figure 6a, the quadrature signals from NH_3_/N_2_ (orange circles) and NH_3_/N_2_ + N_2_O (blue circles) can be seen. From the fitting parameters, fairly good linear trends (*R*_NH_3_/N_2__ = 0.998 and *R*_NH_3_/N_2_+N_2_O_ = 0.999) of the PA signal as a function of the concentration between 0 and 2.0 ppmv were obtained. Besides, the sensitivities (slopes of linear fits) were calculated as slope_(NH_3_/N_2_)_ = (3.0 ± 0.1) μV·ppmv^–1^ and slope_(NH_3_/N_2_+N_2_O)_ = (3.10 ± 0.08) μV·ppmv^–1^. These latter information in conjunction with the standard deviation of the background multiplied by three (3σ_Bg_) – here regarded as the minimal detection threshold – can be utilized to deduce the system lower detection limit (LDL), similarly as did by a contemporary research.^34^ By considering $\sigma_{Bg\left({NH}_{3}/N_{2}\right)}=52\;\mathrm{nV}$ and $\sigma_{Bg\left({NH}_{3}/N_{2}+N_{2}O\right)}=67\;\mathrm{nV}$ the corresponding LDLs of 51 and 67 ppbv were estimated.

**Figure 6:**
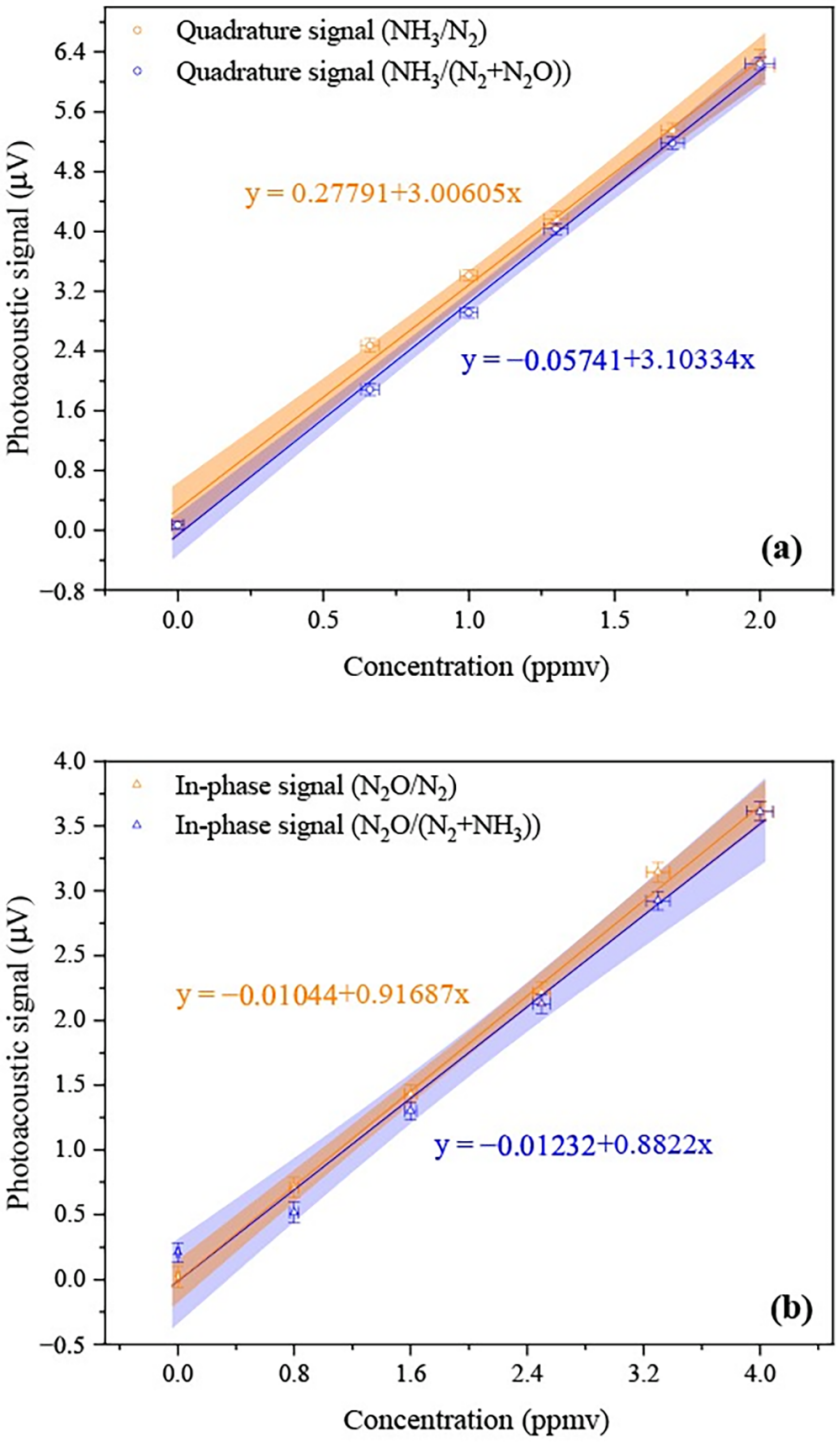
Calibration curves for photoacoustic measurements of NH_3_ (a) and N_2_O (b). Experimental points are shown with horizontal and vertical error bars representing the standard deviations of the gas concentration and photoacoustic signal, respectively. Solid lines denote the best linear fits, and shaded regions indicate the 95% confidence bands of the regression.

In Figure 6b, the in-phase amplitude measurements for N_2_O/N_2_ (represented by orange triangles) and N_2_O/N_2_ + NH_3_ (designated by blue triangles) are illustrated. The data yielded fitting curves that also exhibited strong linear correlations (*R*_N_2_O/N_2__ = 0.999 and *R*_N_2_O/N_2_+NH_3__ = 0.994) of the PA magnitude as a function of concentration within the range of 0–4.0 ppmv. Sensitivity analyzes, analogous to those performed for NH_3_, resulted in sensitivity values of slope_(N_2_O/N_2_)_ = (0.92 ± 0.02) μV·ppmv^−1^ and slope_(N_2_O/N_2_ + NH_3_)_ = (0.88 ± 0.05) μV·ppmv^−1^. Taking into account the standard deviations σ_Bg(N_2_O/N_2_)_ = 80 nV and σ_Bg(N_2_O/N_2_ + NH_3_)_ = 75 nV, the LDLs were calculated to be 262 and 255 ppbv, respectively.

Each calibration point in Figure 6 is presented with both horizontal and vertical error bars, representing the standard deviations of the gas concentration and the photoacoustic signal, respectively. Additionally, the shaded regions surrounding the fitted lines indicate the 95% confidence intervals for linear regression, visually expressing the statistical reliability of the calibration and regression parameters throughout the examined concentration range.

A comparative analysis of the sensitivities provides insight not only into LDLs but also on potential cross-interference effects between NH_3_ and N_2_O. For NH_3_, the sensitivity coefficients ((3.0 ± 0.1) μV·ppmv^–1^and (3.10 ± 0.08) μV·ppmv^–1^) showed a slight difference, well within the range of their respective uncertainties. Similarly, for N_2_O, the sensitivity values displayed ((0.92 ± 0.02) μV·ppmv^–1^and (0.88 ± 0.05) μV·ppmv^–1^) negligible variation. These results suggest that the presence of either analyte in the gas matrix does not give rise to significant cross-talk or mutual interference under the experimental conditions evaluated. The overall calibration trends are robust, thereby confirming the reliability and selectivity of the PA–PRM for independent quantification of NH_3_ and N_2_O.

## Conclusions and Outlook

We introduced a new method for the concurrent sensing of trace gas molecules using photoacoustic (PA) spectroscopy. By inducing a phase difference in the modulation, distinct excitation times are created for NH_3_ and N_2_O, allowing separate absorption spectra via phase-resolved measurements (PRM). The results show no spectral overlap between NH_3_ and N_2_O, enabling independent quasi-simultaneous detection. The calibration curves helped evaluate the behavior and cross-interference between NH_3_ and N_2_O. For NH_3_, linear profiles (*R* = 0.998 and *R* = 0.999) were acquired when N_2_ or N_2_ + N_2_O were adopted as diluting gas. The corresponding sensitivities were (3.0 ± 0.1) μV·ppmv^–1^ and (3.10 ± 0.08) μV·ppmv^–1^, respectively. Using the criterion of the 3σ_Bg_ detection threshold $(\sigma_{Bg\left({NH}_{3}/N_{2}\right)}$ = 52 nV and ${\;\sigma }_{Bg\left({NH}_{3}/N_{2}+N_{2}O\right)}\;$ = 67 nV) the lower detection limits (LDLs) were determined to be 51 and 67 ppbv, respectively. For N_2_O, measurements yielded equally robust calibration data, with regression coefficients of *R* = 0.999 (N_2_O/N_2_) and *R* = 0.994 (N_2_O/N_2_ + NH_3_). Sensitivity analysis produced values of (0.92 ± 0.02) μV·ppmv^–1^and (0.88 ± 0.05) μV·ppmv^–1^, with corresponding LDLs of 262 and 255 ppbv based on $(\sigma_{Bg\left(N_{2}O/N_{2}\right)}$ = 80 nV and $\sigma_{Bg\left(N_{2}O/N_{2}+{NH}_{3}\right)}$ = 75 nV). Comparative analysis of the sensitivities reveals minimal cross-interference effects. For NH_3_, the sensitivity coefficients differed only within their combined uncertainties, while N_2_O sensitivities displayed negligible variation between single-component and binary-mixture scenarios. Error bars representing both gas concentration and PA signal uncertainties, along with 95% confidence intervals for the linear regressions, demonstrate the statistical reliability of the calibration throughout the examined range. These results confirm that the presence of either chemical species does not induce significant cross-talk, thus validating the selectivity and reliability of PRM approach for independent and quasi-simultaneous quantification of NH_3_ and N_2_O in gas mixtures. This technique is relatively easy to implement and offers significant advantages over other methods. It also has potential applications in the study of NH_3_ and N_2_O emissions, especially those related to agriculture involving nitrogen fertilizers.
